# Supplementary material for: SyNDI: synchronous network data integration framework
Source: BMC Bioinformatics. 2018 Nov 6;19:403. doi: 10.1186/s12859-018-2426-5 (PMC6219086; doi:10.1186/s12859-018-2426-5)
Supplement: Supplementary file 5 — Files of the example presented in Additional file 4. This additional file is a zip package that contains all files used in Additional file 4. Detailed descriptions of each of these files are presented in a README file included in this zip package. (GZ 30411 kb) [file 12859_2018_2426_MOESM5_ESM.gz › SyNDITools/Meme2Fimo/meme_4.9.0_4.tar.gz!/meme_4.9.0/website/html/mast-input.html]

MAST - Input


|  |  |
| --- | --- |
|  | MEME Suite The MEME Suite web application requires the use of JavaScript   Javascript doesn't seem to be available on your browser.  MAST -- Motif Alignment and Search Tool  *Motif search tool*   ---   **The input to MAST contains the following fields.**    - **motifs**    This is the name of a file on your computer that contains one   or more of motifs that characterize a group of related sequences.   This will normally be the name of a file that contains the results of a   **MEME** analysis. If you wish to   use motifs from another source it must be first converted to a   compatble format. A "browse" button is   provided by MAST to help you locate the motif file you wish to use.- **treatment of reverse complement strands**      MAST can automatically generate the reverse complement strand for     each **nucleotide** sequence in the database and treat it in     three different ways. ("Given strand" refers to the sequence as it     appears in the database MAST is searching.):     1. **combine with given strand**         MAST searches for motif occurrences on either the given strand or        its reverse complement **together**, not allowing occurrences on        the two strands to overlap each other, and displays them together        as a single sequence. This allows motifs to occur on either strand        and still count toward the overall *E*-value of the        match. (The given strand is the sequence as it appears in the        database MAST is searching.)     2. **treat as separate sequence**         MAST to search for motifs in both the given strand and its reverse        complement, treating them as two, independent sequences. As of version        4.3.2 the results are displayed together in the html though in previous        versions the results were displayed separately for the two strands, as though        both had occurred in the database.     3. **none**         MAST searches only the given strand of each sequence in the        database.     **Note**: this field has no effect when the database contains     protein sequences.   - **use individual sequence composition in *E*-and     *p*-value** calculation      This option can improve search selectivity when erroneous matches     are due to **biased sequence composition**. MAST normally     computes *E*-values and *p*-values using a random     sequence model based on the **overall** letter composition of     the **database** being searched. Selecting this option will     cause MAST to use a **different** random model for **each     target sequence**. The random model for each target sequence will     be based on its letter composition, not that of the entire     database. Using this option will tend to give more accurate     *E*-values and increase the *E*-values of compositionally     biased sequences. This option may **increase search times     substantially** if used in conjunction with *E*-value display thresholds over 10, since MAST     must compute a new set of motif score distributions for each     high-scoring sequence.   - **ignore motifs with high *E*-values**      MAST can ignore motifs in the query with *E*-values above a     threshold you select. This is desirable because motifs with high     *E*-values are unlikely to be biologically significant. The     default threshold will cause MAST to use **all** motifs in the     query, regardless of their *E*-values.      **Note:** This option is only available for motifs generated by     MEME 3.0 and above.   - **search nucleotide database with protein motifs**      Choosing this option will cause MAST to search the nucleotide     version of the selected sequence database, converting the     nucleotide sequences to protein sequences in all six reading     frames. By default, MAST searches the protein version of the     selected database when you give it a file of protein motifs.   - **scale motif display threshold by sequence length**      MAST displays motifs that score above a threshold for all     high-scoring sequences. By default, this threshold is based on the     probability of the motifs without regard to the length of the     sequence. The threshold was chosen with protein sequences of     average length in mind. Consequently, many positions in very long     sequences may match motifs with scores above this threshold by     chance, making the results difficult to interpret. Selecting this     option causes the motif display threshold to take sequence length     into account. This will reduce the number of weak motifs displayed     in long sequences and minimize the size of the output file.   - ***E*-value display threshold**      MAST only displays sequences matching your query with     *E*-values below the given threshold you specify here. By     default, sequences in the database with matches with     *E*-values less than 10 are displayed. If your motifs are very     short or have low information content (are not very specific), it     may be impossible for any sequence to achieve a low *E*-value.     If your MAST search returns no hits, you may wish to increase the     *E*-value display threshold and repeat the search.  **Search using MAST**  **MAST introduction**  **MEME SUITE introduction** |
